# Impact on patient outcomes of spondyloarthritis-inflammatory bowel disease multi-disciplinary meetings

**DOI:** 10.1093/rheumatology/keae116

**Published:** 2024-02-23

**Authors:** Sarah Sayers, Danielle Lam, Qutab Shah, Jobie Evans, Miles Parkes, Carmel Stober, Joanne Rimmer, Gavin Clunie, Tania-Elena Gudu, Denise Rosembert, Sreedhar Subramanian, Stephanie Brookes-Jones, Stephen Moss, Tim Raine, Deepak Jadon

**Affiliations:** Rheumatology Research Unit, Department of Medicine, University of Cambridge, Cambridge, UK; Department of Gastroenterology, Cambridge University Hospitals NHS Foundation Trust, Cambridge, UK; Rheumatology Research Unit, Department of Medicine, University of Cambridge, Cambridge, UK; Rheumatology, Addenbrooke's Hospital, Cambridge, UK; Department of Gastroenterology, Cambridge University Hospitals NHS Foundation Trust, Cambridge, UK; Department of Gastroenterology, Cambridge University Hospitals NHS Foundation Trust, Cambridge, UK; Department of Gastroenterology, Cambridge University Hospitals NHS Foundation Trust, Cambridge, UK; Department of Gastroenterology, Cambridge University Hospitals NHS Foundation Trust, Cambridge, UK; Department of Gastroenterology, Cambridge University Hospitals NHS Foundation Trust, Cambridge, UK; Department of Gastroenterology, Cambridge University Hospitals NHS Foundation Trust, Cambridge, UK; Department of Gastroenterology, Cambridge University Hospitals NHS Foundation Trust, Cambridge, UK; Department of Gastroenterology, Cambridge University Hospitals NHS Foundation Trust, Cambridge, UK; Department of Gastroenterology, Cambridge University Hospitals NHS Foundation Trust, Cambridge, UK; Department of Gastroenterology, Cambridge University Hospitals NHS Foundation Trust, Cambridge, UK; Rheumatology Research Unit, Department of Medicine, University of Cambridge, Cambridge, UK

**Keywords:** multidisciplinary, spondyloarthritis, inflammatory bowel disease, Crohn’s disease, ulcerative colitis

## Abstract

**Objectives:**

To assess the impact on patient outcomes of the spondyloarthritis (SpA) and inflammatory bowel disease (IBD) multidisciplinary team (MDT) meetings in a large university hospital.

**Methods:**

A single-centre retrospective observational case-note review was conducted assessing the outcome of all 226 cases discussed at the SpA–IBD MDT meetings in a large UK university hospital between 2017 and 2022.

**Results:**

A total of 226 patients were discussed. It was deemed that 97% of MDT meetings helped to improve communication between teams, and 100% were educational. A total of 57% of discussions led to an instant change of disease management, while 40% of discussions resulted in a treatment plan that avoided the use of dual advanced therapy. This improved patient safety by reducing immunosuppression. The MDT meetings were highly cost and time efficient; 125 referrals between specialists were avoided, and in 51 cases there was a significant chance of reducing future drug costs. A timely investigation or appointment was arranged following 50% of MDT discussions, helping to clarify the diagnosis and optimize patient care. Nine percent of meetings enabled drugs to be prescribed to patients that are not yet licensed for the other speciality, thereby improving treatment options available in the management of complex cases.

**Conclusion:**

The MDT meetings have been beneficial for patients, the clinical team and the institution. This approach might be considered by other rheumatology and gastroenterology departments.

Rheumatology key messages

- MDT meetings are beneficial for patients, the clinical team and the institution.
- An instant change to disease management was commonplace, and many referrals between specialists were avoided.
- High-cost drug use was reduced, and differential licencing enabled earlier access to advanced therapies.

## Introduction

Spondyloarthritis (SpA) is a heterogeneous group of auto-inflammatory rheumatic diseases that cause musculoskeletal inflammation. The spondyloarthritides includes ankylosing spondylitis (a.k.a. radiographic axial spondyloarthritis), non-radiographic axial spondyloarthritis, peripheral spondyloarthritis, reactive arthritis, psoriatic arthritis and enteropathic arthritis. Whilst these conditions primarily affect the musculoskeletal system, non-musculoskeletal manifestations such as psoriasis, uveitis and inflammatory bowel disease (IBD) are often present [1]. IBD includes ulcerative colitis and Crohn’s disease, which are characterized by chronic inflammation of the gastrointestinal tract [2].

IBD and SpA have some shared pathogenesis, involving the so-called ‘gut–synovial axis’, which implicates genetic, environmental and host factors in the initiation and perpetuation of inflammation [1, 3]. A few specific human leucocyte antigens (HLA) alleles, e.g. *HLA-B27*, *HLA-B35* and *HLA-B44*, increase the likelihood of patients with IBD developing SpA, and associations with IL-23, TNF-α and α4β7 integrin have also been implicated in the pathophysiology of the gut–synovial axis [1]. These proteins are the target of several biologic and targeted synthetic drugs, which are licensed for use in IBD and SpA. The shared pathophysiology highlights the complexity and challenge of patient management, and emphasizes the role of multidisciplinary and multi-speciality working to optimize care [4, 5].

A multidisciplinary team (MDT) is a group of health care professionals from different fields who work together to share their expertise to improve treatment efficiency and patient care [6]. This approach first emerged in the field of oncology but has recently been used and recommended in the treatment of other complex conditions, such as IBD and SpA.

Our group have been piloting multidisciplinary management in the care of patients with IBD and SpA. In 2017, an IBD–SpA MDT was formed, consisting of rheumatology and gastroenterology consultants, fellows, specialist and research nurses, and a biologic pharmacist. The minimum requirement for the MDT was the attendance of at least one consultant gastroenterologist and one consultant rheumatologist (usually two to three physicians attended from each speciality); cases were pre-submitted and presented by a clinical fellow, who then summarized recommendations and actions to MDT contributors.

In a series of meetings every 2 months, these complex patients were discussed and the team sought to share their diverse expertise and knowledge to work together, broaden the team’s knowledge and skills, and to improve patient management.

## Methods

A retrospective single-centre observational case-note review was performed, as part of a service evaluation, to assess the outcome of all patients discussed in the 2-monthly SpA–IBD MDT meetings in a large university hospital between January 2017 and December 2022.

This was a service improvement project (SIP) in which we audited clinical practice within our department and compared with the international GRAPPA, ASAS and NICE SpA clinical standards and recommendations on the management of SpA. The SIP was reviewed and registered with the Cambridge University Hospitals NHSFT audit department. We used the online UK REC tool to determine that this was not a research project, and therefore did not require ethical (REC) approvals.

Patients with an established diagnosis of SpA and/or IBD were referred to the MDT by their Rheumatology or Gastroenterology consultant, or via a specialist nurse. This included patients with an established diagnosis of either SpA or IBD, who had clinical symptoms suggestive of IBD or SpA, respectively. Patients who had concomitant IBD and SpA at the time of discussion were referred due to inadequate control of one or both diseases. The occurrence of the bimonthly SpA–IBD MDT was publicized to all rheumatology and gastroenterology consultants, such that patients could also be referred from general clinics.

Data were collected from the hospital’s electronic patient record system (EPIC), including the MDT notes, doctors’ letters and other entries. To improve impartiality regarding patient outcomes, data were collected by a single medical student (S.S.), with clinical mentorship by a senior rheumatology fellow (Q.S.) who has not been involved in the MDTs.

**Figure 1.**
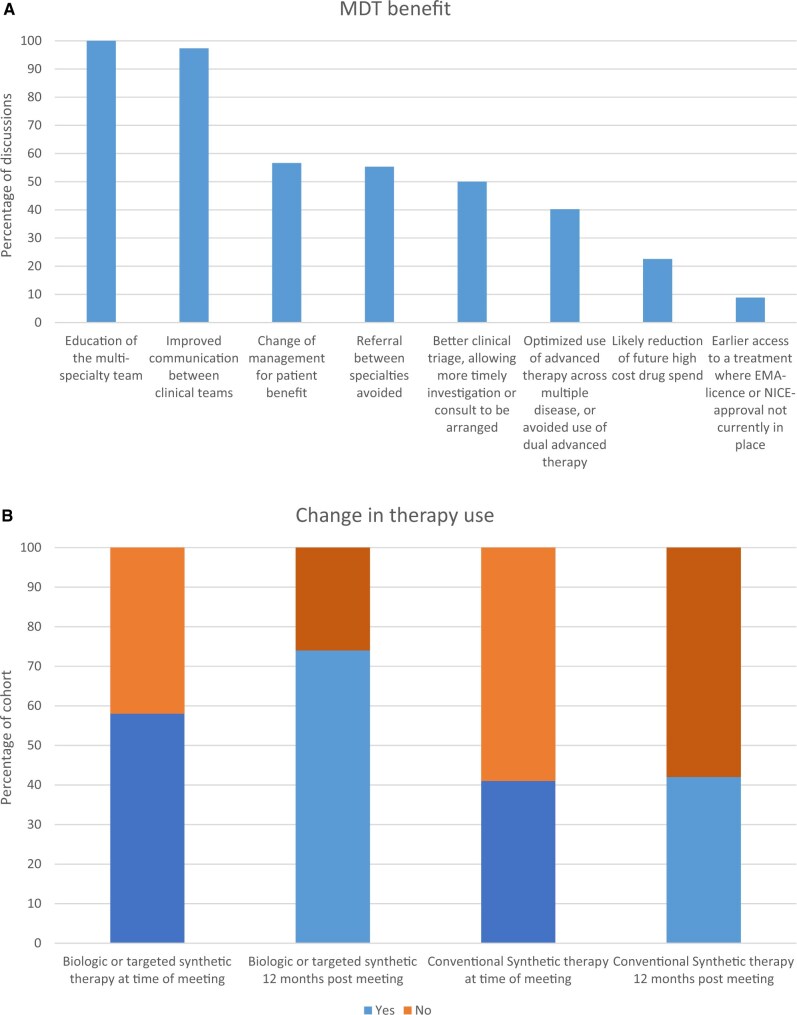
(A) Clinical outcomes from the multidisciplinary team (MDT) meetings. (B) Graph showing the change in therapy use

A structured data collection sheet was devised and comprised fields relating to: date of MDT discussion; department referring the case; patient characteristics (age, sex, BMI, smoking history); relevant comorbidities and related conditions; and immunomodulator therapy use (conventional synthetic, biologic or targeted synthetic) of the patient at the time of the meeting and at clinical follow-up 12 months later. Follow-up was assessed by reviewing patients’ clinical notes at ∼12 months post-discussion; there was no routine referral back to the MDT. Follow-up data were available for 197/226 cases. Data on the following clinical management parameters were collected: the reason for referral to the meeting (such as a referral request for clinical assessment; general advice from the multi-speciality team; how to optimize patient management). For homogeneity, nine different potential benefits of the MDT were assessed: change of therapeutic management; patient safety (including the avoidance of the concomitant prescription of two advanced therapies); earlier access to a safer treatment due to differential licencing; timely investigation or consult arranged; referral between specialities avoided; likely reduction of high-cost drug spend in the future; recruitment to a clinical trial; improved communication between teams; and clinical education of the MDT (Fig. 1).

## Results

### Demographic and clinical characteristics

Between May 2017 and November 2022 a total of 34 IBD SpA MDT meetings took place, with 226 cases being discussed, comprising 163 unique patients (Table 1). The median age was 49 [interquartile range (IQR) 39–56; range 17–78], 67% were female, 55% were ever-smokers, 68% were classified as having a BMI >25 (mean BMI 29.01) and only 28% had a normal BMI (20–25).

**Table 1.** A summary of data collected from the IBD–SpA MDT meetings

Data collected	Value
Number of cases discussed	226
Number of unique cases	163
Clinical characteristics	
Age, mean, years	48.36
Female, *n* (%)	152/226 (67)
Ever smoker, *n* (%)	120/219 (55)
BMI, *n* (%)	
<20	8/224 (4)
20–25	63/224 (28)
25–30	70/224 (31)
30–35	38/224 (17)
35–40	35/224 (16)
40–50	10/224 (4)
Median (IQR)	28.07 (23.76–34.26)
Diagnosis	
IBD, *n* (%)	
Crohn’s disease	145/226 (64)
Ulcerative colitis	54/226 (24)
Other	14/226 (6)
Total	213/226 (94)
SpA, *n* (%)	
AxSpA	53/226 (23)
PsA	28/226 (12)
Enteropathic arthritis	119/226 (53)
Other	38/226 (17)
Total	220/226 (97)
Comorbidity, *n* (%)	
Chronic pain	13/226 (6)
Dyslipidaemia	5/226 (2)
Depression	19/226 (8)
Hypertension	28/226 (12)
Fatty liver disease	6/226 (3)
Gout	8/226 (4)
Number of comorbidities, *n* (%)	
1	8/226 (4)
2	24/226 (11)
3	49/226 (22)
4	61/226 (27)
5	48/226 (21)
6	28/226 (12)
7	6/226 (3)
8	2/226 (1)
Therapeutic management, *n* (%)	
Biologic or targeted synthetic therapy at the time of SpA–IBD MDT meeting	131/226 (58)
Biologic or targeted synthetic therapy 12 months after SpA–IBD MDT meeting	145/197 (74)
Conventional synthetic therapy at the time of SpA–IBD MDT meeting	93/226 (41)
Conventional synthetic therapy 12 months after SpA–IBD MDT meeting	83/197 (42)
Referring speciality, *n* (%)	
Gastroenterology	106/226 (47)
Rheumatology	106/226 (47)
Joint referral	14/226 (6)
Reason for case discussion, *n* (%)	
Therapeutic query	166/266 (73)
Requesting a clinical consult	57/226 (25)
Seeking general/diagnostic advice	84/226 (37)
Beneficial outcome for which speciality, *n* (%)	
Gastroenterology	56/218 (26)
Rheumatology	121/218 (56)
Both	41/218 (19)
Benefit, *n* (%)	
Change of management for patient benefit	128/226 (57)
Optimized use of advanced therapy across multiple diseases, or avoided the use of dual advanced therapy	91/226 (40)
Earlier access to a treatment where EMA-license or NICE-approval not currently in place	20/226 (9)
Likely reduction of future high-cost drug spend	51/226 (23)
Better clinical triage, allowing more timely investigation or consult to be arranged	113/226 (50)
Referral between specialities avoided	125/226 (55)
Improved communication between clinical teams	220/226 (97)
Education of the multi-speciality team	226/226 (100)

AxSpA: axial SpA; IQR: interquartile range; MDT: multidisciplinary team.

At the time of discussion, 213/226 (94%) had a diagnosis of IBD (145 Crohn’s disease and 54 ulcerative colitis). A diagnosis of SpA was present in 220/226 (97%) [53 axial spondyloarthritis (axSpA), 28 psoriatic arthritis (PsA) and 139 other forms of inflammatory arthritis, such as enteropathic arthritis]. A total of 131/226 (58%) were on biologic or targeted synthetic therapy, and 93/226 (41%) on conventional synthetic drugs to treat their IBD and/or SpA. The number of comorbidities ranged between 1 and 8, with a median of 4, and included: inflammatory bowel disease, mesenteric panniculitis, ischaemic colitis, coeliac disease, diverticulitis, rheumatoid arthritis, osteoarthritis, Still’s disease, fibromyalgia, gout, chronic pain, hypertension, dyslipidaemia, ischaemic heart disease, fatty liver disease and depression.

The two specialities referred an equal number of cases to the meetings over the 5-year period. The main reasons for referral to the SpA–IBD MDT were: advice on management (73%); request to refer the patient to the other speciality (25%); and to aid the diagnostic process (37%).

### Patient benefits

Patients benefitted from being discussed in SpA–IBD MDT meetings. A change in patient management, such as changing an immunomodulator or initiating physiotherapy, occurred following 128/226 (57%) discussions. Following the discussion, 20 (9%) patients commenced novel therapies that at the time of prescribing were only licensed for use in one speciality, but were under late phase clinical development in indications for the other speciality, thereby improving the treatment options available for complex cases. A total of 91/226 (40%) discussions related to the need for advanced immunomodulator therapy for both IBD and SpA, and MDT discussion often permitted the recommendation of a single advanced immunomodulator therapy designed to target both conditions. This optimized efficacy across several diseases, such as including skin psoriasis and uveitis, in addition to IBD and SPA, and avoided exacerbation of a related condition, e.g. prescription of IL-17i for SpA at the detriment of the IBD. Thus, it improved patient safety by avoiding the accidental prescription of two different advanced immunomodulators.

Conversely, the SpA–IBD MDT led to the establishment of a more refined biologic prescribing policy, enabling a few of the most complex patients to receive dual biologic therapy. In total, six (4%) patients benefited directly from this.

One year following the case discussion, 145/197 (74%) patients were on biologic or targeted synthetic therapy, and 83/197 (42%) were on conventional synthetic therapy (Fig. 1). This cohort represents individuals with severe IBD and/or SpA, so the change in biologic therapy use from 58% at baseline, to 74% after one year represents a modest increase.

One patient was recruited to a clinical trial following an SpA–IBD MDT, but two patients were removed from a trial, due to the need to change an advanced therapy.

### Institutional benefits

A reduction in future high-cost advanced immunomodulator therapy expenditure was deemed to occur in 51/226 (23%) cases. This was awarded to any patient who at 12-month follow-up had optimized their therapeutic management without receiving a biologic or targeted synthetic therapy. In addition, 125 referrals to the gastroenterology or rheumatology departments were avoided due to the discussions. Following 113/226 (50%) discussions, the receiving clinician was better able to appreciate and triage the patient for prompt investigation or consult, thereby enabling a more rapid diagnosis and initiation of management.

### Clinical team benefits

The SpA–IBD MDT also demonstrated an improvement in communication between the rheumatology and IBD clinical teams. In particular the nuances of the clinical presentation were more effectively communicated verbally rather than in the form of written correspondence. A ‘personalized’ familiarity with the patient before their attendance to the outpatient consult can aid the rapport and clinician–patient interaction.

It was considered that 100% of the meetings helped to educate the multi-speciality team on IBD and rheumatology considerations in the care of patients (Table 1). Educational talking points included: epidemiology; clinical presentation; subphenotypes of disease; natural history of disease and prognostic markers; established and novel diagnostic approaches; dose titration of advanced immunomodulators; utility of serum drug level and anti-drug antibody testing; latest therapeutic advances; horizon scanning of soon to be EMA-licensed or NICE-approved medications; international management recommendations and national policy on healthcare provision through the experience of clinicians sitting on such national and international committees; and inclusion of allied health care professionals to support holistic non-pharmacological interventions.

## Discussion

MDT meetings as based upon the premise that collective decision making, in which clinicians from a variety of specialities share knowledge and expertise to systematically discuss a complex case, enables optimal patient management. They are considered a gold standard approach of patient management for individuals with IBD or SpA [7, 8]. Whilst recent reviews advocate this approach, there is little published evidence demonstrating its effectiveness. This retrospective single-centre observational service evaluation outlines the benefits that MDTs offer.

The MDT discussions often led to a change in patient management, in particular to advanced therapies (biologic and targeted synthetic therapy) that would address more facets of a patient’s disease, e.g. Crohn’s disease, peripheral and axial arthritis, skin psoriasis and/or inflammatory eye disease. Many patients were able to access consults, investigations, physiotherapy and other non-pharmacological therapies earlier than they would have been able to through standard pathways and written communication. Two clinical trial patients were removed following extensive discussion in the meeting, and one patient was recruited to a different trial.

The meetings provide an opportunity for clinicians to learn from each other and understand more about their own speciality and the other. Learning in this team environment has enabled connections to be fostered between health care professionals in different specialities, improving communication within and between different departments.

Such discussions were academically stimulating, identified areas of unmet clinical need and thus led to the inception, research grant award, appointment of an MD research fellow, and completion of a 2-year academic study investigating the prevalence and utility of existing magnetic resonance enterograms to identify undiagnosed SpA in a cohort of patients with Crohn’s disease (ProSpA-CD study; https://clinicaltrials.gov/ct2/show/study/NCT03817983). These data are currently being analysed, and the results are expected to benefit patient care through novel disease screening strategies and healthcare provision pathways.

The institution benefited directly from a more targeted and considered use of resources in this patient population. Receiving clinicians were better able to appreciate and triage the patient for prompt investigation or consult, thereby enabling a more rapid diagnosis and initiation of management.

The inclusion of every case that has been discussed in the IBD-SpA MDT meetings over a 5-year period is a strength of this study, as it reduces selection bias. Data was collected and analysed by two individuals (S.S. and Q.S.) who had not been involved in the MDTs, and therefore were less biased in its favour. The study would have been improved by more data on medication use and clinical disease activity indices at the time of the initial discussion and 12 months later. Due to the retrospective nature of this study, and also the heterogeneous patient cohort, standardized outcome measures were unfortunately not designed or collected at the time of the case discussions or at the 12-month follow-up period. Rather, postulated benefits were retrospectively discussed by individuals who had been present at the meetings, and clinic notes were reviewed by S.S. and Q.S. to assess whether they had been achieved. Furthermore, the absence of a control group is a clear limitation. The regular collection of patient-reported outcome measures may have facilitated obtaining a comparator for those patients who were not referred to the SpA–IBD MDT, although it should be recognized that more complex cases are likely to be referred.

The format of the SpA–IBD MDT meetings comprised the gastroenterology and rheumatology teams meeting, but the patient was absent. The recommendations of the MDT were subsequently discussed with patients by telephone, at the next consult or by letter. Their input on further management was accordingly sought. However, contemporaneously involving patients in the discussions would have added further value, as unknown clinical information may have been gleaned, and clinical reasoning might have been framed by the patients’ perspective on matters. There are alternatives to the MDT meeting approach practiced in our institution. Alternatives include ‘combined clinics’ or ‘group consultations’, in which the patient consults several members of a clinical team in a coordinated manner [4]. The delivery of such models can be logistically and financially more challenging than our current model, but they are certainly worth exploring for selected complex patients. A further study might compare patient satisfaction in departments that have adopted combined clinics with that where patients are discussed in MDT meetings.

## Conclusion

To conclude, this study has identified several benefits from hosting regular SpA–IBD MDT meetings. Patients, doctors and the institution have all greatly benefited from these discussions to a great extent. Other gastroenterology and rheumatology departments might consider implementing a similar multidisciplinary and multi-speciality approach.

## Data Availability

The data underlying this article will be shared at aggregate/population level on reasonable request to the corresponding author. Patient level data underlying this study cannot be made available to researchers.
